# Identifying distinctive brain regions related to consumer choice behaviors on branded foods using activation likelihood estimation and machine learning

**DOI:** 10.3389/fncom.2024.1310013

**Published:** 2024-01-17

**Authors:** Shinya Watanuki

**Affiliations:** Department of Marketing, Faculty of Commerce, University of Marketing and Distribution Sciences, Kobe, Hyogo, Japan

**Keywords:** brand equity, consumer neuroscience, neuromarketing, multi-voxel pattern analysis, multi-coordinate pattern analysis, sPLS-DA

## Abstract

**Introduction:**

Brand equity plays a crucial role in a brand’s commercial success; however, research on the brain regions associated with brand equity has had mixed results. This study aimed to investigate key brain regions associated with the decision-making of branded and unbranded foods using quantitative neuroimaging meta-analysis and machine learning.

**Methods:**

Quantitative neuroimaging meta-analysis was performed using the activation likelihood method. Activation of the ventral medial prefrontal cortex (VMPFC) overlapped between branded and unbranded foods. The lingual and parahippocampal gyri (PHG) were activated in the case of branded foods, whereas no brain regions were characteristically activated in response to unbranded foods. We proposed a novel predictive method based on the reported foci data, referencing the multi-voxel pattern analysis (MVPA) results. This approach is referred to as the multi-coordinate pattern analysis (MCPA). We conducted the MCPA, adopting the sparse partial least squares discriminant analysis (sPLS-DA) to detect unique brain regions associated with branded and unbranded foods based on coordinate data. The sPLS-DA is an extended PLS method that enables the processing of categorical data as outcome variables.

**Results:**

We found that the lingual gyrus is a distinct brain region in branded foods. Thus, the VMPFC might be a core brain region in food categories in consumer behavior, regardless of whether they are branded foods. Moreover, the connection between the PHG and lingual gyrus might be a unique neural mechanism in branded foods.

**Discussion:**

As this mechanism engages in imaging the feature-self based on emotionally subjective contextual associative memories, brand managers should create future-oriented relevancies between brands and consumers to build valuable brands.

## 1 Introduction

Building brands that consumers associate with strong values, which are referred to as “brand equity,” is imperative for building profitable enterprises (Keller, 1993; Aaker, 1996). Consumers’ choice behavior related to brand equity is underlain by the complex mental processes woven by rational and emotional cognitive systems. According to Aaker, brand equity comprises five elements: brand awareness, brand association, brand loyalty, perceived quality, and proprietary assets (Aaker, 2009). Keller insisted that brand knowledge is the most important element of brand equity (Keller, 1993). Since brand equity can be considered a set of memories in consumers’ minds (Kapferer, 2008), these memories might be the most crucial element of brand equity. The types of memories crucial for building brand equity as well as the information and mental processes in consumers’ minds have been an important focus of research. Moreover, many studies in consumer neuroscience have investigated brain regions related to brand equity. McClure et al. (2004) demonstrated that both the dorsal lateral prefrontal cortex (DLPFC) and hippocampus are characteristic brain regions related to brand equity, based on an experiment inspired by the famous Pepsi challenge. Given that their findings were consistent with previous marketing literature (Keller, 1993; Aaker, 1996), they concluded that episodic memory derived from the hippocampus might be a key differentiator between brands with high and low brand equity. However, Deppe et al. (2005) demonstrated intensive activation of the ventral medial prefrontal cortex (VMPFC) compared with the DLPFC in the case of branded food products. In non-food categories, several brain regions, such as the medial prefrontal cortex from the ventral to orbital regions, posterior cingulate cortex, and striatum, were activated by the Apple brand logo stimulus (Murawski et al., 2012) and luxury brand products (Audrin et al., 2017). In previous studies on global high-reputation brands, Yoon et al. (2006) demonstrated distinctive activation of the left inferior frontal gyrus (IFG), whereas Chen et al. (2015) demonstrated activation of a wide variety of brain regions (medial prefrontal cortex, posterior regions, parietal regions, and striatum), including the IFG. Thus, the findings on unique brain regions associated with brand equity remain controversial.

Neuroimaging meta-analytical methods have prevailed in revealing brain regions related to specific mental processes by aggregating many studies related to the research objectives (Wager et al., 2004; Eickhoff et al., 2009; Radua and Mataix-Cols, 2009; Salimi-Khorshidi et al., 2009). However, identifying the extent to which a particular brain region contributes to mental processes related to the choice behaviors on branded products using only neuroimaging meta-analytic methods is challenging. Multi-voxel pattern analysis/multivariate pattern analysis (MVPA) is an appropriate approach to explicitly uncover brain regions contributing to particular mental processes by predicting cognitive function based on the voxel data of activated neuroimages using machine learning techniques (Norman et al., 2006; Haxby, 2012). Because the linear support vector machine (l-SVM) algorithm is used for MVPA in many cases, observed voxel patterns are set as features, and mental processes are set as outcomes. Thus, the intensities contributing to mental processes are calculated as particular voxel patterns in all brain regions. MVPA enables researchers to rigorously infer cognitive functions using a data-driven approach without arbitrary inferences. Ariely and Berns (2010) suggested that applying MVPA to consumer neuroscience and neuromarketing might be effective in revealing hidden information about consumers’ minds in purchase behavior Several studies have reported the application of MVPA to consumer choice behavior. The broad regions of the medial prefrontal cortex and insula have been shown to contribute to predicting car preferences (Tusche et al., 2010). Another study showed that activated patterns derived from a healthy package design in the medial superior frontal gyrus and middle occipital gyrus were significant distinctive brain regions for predicting food choices (Van der Laan et al., 2012). Similarly, Pogoda et al. (2016) investigated the predictive brain regions for daily confectionery categories sold in stores. They revealed that broad regions of the MPFC, DLPFC, and dorsal anterior cingulate cortex were distinctive brain regions for predictions. These results suggest that the contributions of brain regions for predicting choice behaviors might depend on product involvement. The dorsal part of PFC was observed as the brain region contributing to predictions for choice behaviors in studies using confectionary categories (low involvement products) as an experimental stimulus but not in those using cars (high involvement products). The consumer information process theory states that the types of consumer information processes depend on product involvement (Kollat et al., 1972). Although MVPA is useful for revealing the contribution of particular brain regions to mental processes, no studies have applied MVPA to neuroimaging meta-analytical methods.

Therefore, in this study, we limited our focus area to the food category (low involvement categories). We aimed to clearly identify the contributing brain regions to branded food choice behavior using neuroimaging meta-analytical methods and machine learning techniques.

## 2 Materials and methods

We adopted a neuroimaging meta-analytical method and machine learning to uncover characteristic brain regions related to brand equity in comparison with consumers’ decision-making between branded and unbranded food products. The former approach aims to reveal the shared and distinctive brain regions affecting consumer choice behavior between branded and unbranded foods by comprehensively gathering neuroimaging studies. Meanwhile, the latter can classify and predict types of choice behaviors, whether branded or not, based on the brain regions observed using neuroimaging methods.

### 2.1 Meta-analytical neuroimaging method

The neuroimaging meta-analytical method is used to determine activation of brain regions related to cognitive functions and diseases by gathering related studies using procedures subjected to the standard guidelines. This method involves two major approaches: image-based meta-analysis (IBMA) and coordinate-based meta-analysis (CBMA). Because IBMA uses actual neuroimaging data, activated brain regions related to research objectives can be accurately revealed. IBMA is superior to CBMA because it uses massive amounts of information on activated brain regions with a fully statistically brain-activated brand map (Salimi-Khorshidi et al., 2009). However, this approach faces major challenges in gathering imaging data, as data from old studies could be lost, and contacting researchers who may be able to source this data is difficult. In contrast, the CBMA has no challenges with regards to data collection. Although CBMA might lose this information compared with IBMA, CBMA is highly accessible to researchers because it can use published studies for analyzing activated brain regions. The CBMA approach can produce commonly activated brain regions related to research objects, based on the foci reported in published papers. The accessibility of CBMA has increased its utility as a neuroimaging meta-analysis method. Three major calculation methods have been developed for CBMA: activation likelihood estimation (ALE) (Eickhoff et al., 2009), multi-kernel density analysis (MKDA) (Wager et al., 2004), and signed differential mapping (SDM) (Radua and Mataix-Cols, 2009). Because the ALE was validated in comparison with the IBMA, activated brain maps of the ALE had higher correlation coefficients with those of the IBMA than with other CBMA approaches (Salimi-Khorshidi et al., 2009). Therefore, the ALE might be a preferable method among CBMA approaches, and we adopted the ALE method to calculate the activated brain regions in our meta-analysis.

#### 2.1.1 Calculating activated brain regions using ALE

**Figure 1:**
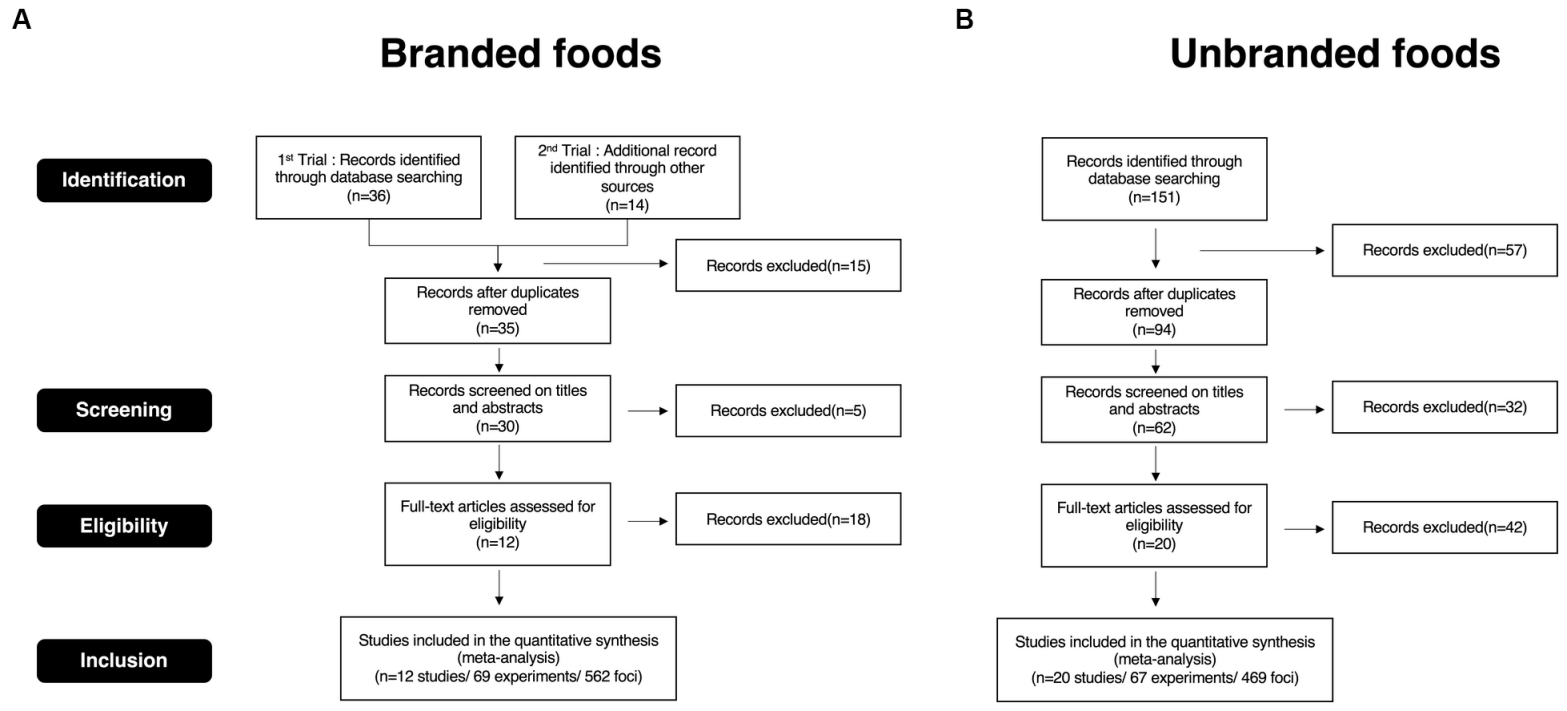
PRISMA flow diagram. **(A)** PRISMA flow diagram for branded foods studies selection. **(B)** PRISMA flow diagram for unbranded foods studies selection.

First, we collected appropriate publications according to the Preferred Reporting Items for Systematic Reviews and Meta-Analyses (PRISMA) guidelines. A PRISMA flow diagram is shown in Figure 1. We searched studies related to our research using several search words from the Pubmed database (https://pubmed.ncbi.nlm.nih.gov). Search words for brand equity-related studies were as follows: “brand, fMRI, neural, and choice,” “brand, fMRI, neural, and purchase,” “brand, fMRI, neural, and decision-making,” and “brand, fMRI, neural, and preference.” Search words for un-branded objects-related studies, which are studies focused on consumer behavior in general decision-making regardless of whether the objects were branded or not were as follows: “consumer, fMRI, neural, and choice,” “consumer, fMRI, neural, and purchase,” “consumer, fMRI, neural, and decision-making,” and “consumer, fMRI, neural, and preference.” The other regulations for selecting the studies were as follows: We included studies published between January 2000 and March 2023. All publications adopted for this meta-analysis were written in English and peer-reviewed in international journals. Moreover, Plassmann’s list (Plassmann et al., 2012), a well-known consumer neuroscience study selection for brand equity research, was added as an additional data resource for searching for branded studies. We excluded studies that matched the following conditions in the screening phase for title and abstract, as well as duplicate studies: (1) meta-analysis; (2) review articles; (3) studies without magnetic resonance imaging (MRI) data; (4) disease studies; (5) other non-consumer context studies; and (6) non-food research objective. During the eligibility phase, we checked the following items in addition to those checked in the screening phase: (1) use of food-related stuff as an experiment stimulus; (2) use of a brand logo as an experimental stimulus in assessing brand; (3) report of coordinates in activated brain regions; and (4) reported foci described in the three-dimensional stereotactic space of the Talairach or Montreal Neurological Institute (MNI). Although a brand logo was not used as an experimental stimulus by Plassmann et al. (2008), we adopted this study for this meta-analysis for two reasons. First, this study was listed as a branded study in the well-known consumer neuroscience study selection (Plassmann et al., 2012). Second, the research objectives and findings of this study could be considered a cognitive function of brand association in uncertain situations. For the present study (Supplementary Tables S1A,B), 12 studies (562 foci) were included in the branded foods group, whereas 20 studies (469 foci) were included in the unbranded foods group.

ALE is a calculation method for seeking peak coordinates in activated brain regions by applying the maximum likelihood estimation method to a quantitative neuroimaging meta-analysis. The overall calculation procedure is as follows (Turkeltaub et al., 2012; Fox et al., 2014):

1. Modeled activation maps were produced by applying a Gaussian probability density function to each focus (Eq. 1).

(1) $$L_{ik}\left(\nu \right)=c\phi_{3}\left(\nu |x_{ik},\sigma_{i}^{2}I\right)$$

ν is a voxel, and $x_{ik}$ represents the reported focus. As for study *i*, $L_{ik}$ is the map corresponding with a single $x_{ik}$ , and $\phi_{3}\left(x\mathrm{;,}\mu \mathrm{;,\Sigma}\right)\;$ is a three-dimensional Gaussian probability density function. The parameters of both μ and Σ are the mean and covariance matrix, respectively. I is the identity matrix, and c is a constant coefficient for transforming the sum of $\phi_{3}\left(.\right)$ over voxel into one.


(2) $$MA_{i}\left(\nu \right)=\underset{k}{\max}L_{ik}\left(\nu \right)$$

$MA_{i}$ is a modeled activation map (Eq. 2). $MA_{i}$ is also an individual map of maximum activation likelihood.

1. An ALE map was created by gathering and uniting the modeled activation maps (Eq. 3).

(3) $$ALE\left(\nu \right)=1-\overset{I}{\underset{i=1}{\prod }}MA_{i}\left(\nu \right)$$

ALE(ν) represents an ALE value, which is the probability that might be the closest activated locations over all foci.

1. The thresholded ALE map was obtained by conducting a permutation test between each voxel in the ALE map and that in the randomness map based on the null distribution. ALE values based on the null distribution are referred as to the null ALE (Eq. 4). Thus, the more foci that are gathered and converged, the more accurately the activated brain regions can be calculated.

(4) $$ALE^{*}\left(\nu \right)=1-\overset{I}{\underset{i=1}{\prod }}MA_{i}\left(\nu^{*}\right)$$

$ALE^{*}\left(\nu \right)$ is the null ALE—an ALE value randomly calculated from each activated map based on random locations. $\nu^{*}$ represents a voxel randomly sampled from the null distribution.

We calculated the ALE algorithm using the GingerALE version 3.0.2 software.^1^ The parameters for calculating the ALE algorithm were as follows: (1) cluster-level correction for multiple comparisons, *p* = 0.05; (2) cluster-forming threshold, *p* = 0.001; and (3) permutation size: 1000. The obtained branded and un-branded ALE maps were produced as NIfTI files and visualized using MANGO version 4.1 software.^2^

### 2.2 Machine learning technique

As described earlier, MVPA using machine learning techniques has been widely used in the neuroscience field to identify and classify brain regions related to cognitive function. However, unlike imaging data of activated brain regions, even if the coordinates data from each study in a CBMA could be obtained, applying the data-driven approach to this study is challenging without any devices. Thus, we conducted feature engineering on the raw coordinate data. In this study, we refer to the proposed predictive method using coordinate data as multi-coordinate pattern analysis (MCPA).

#### 2.2.1 Feature engineering

**Figure 2:**
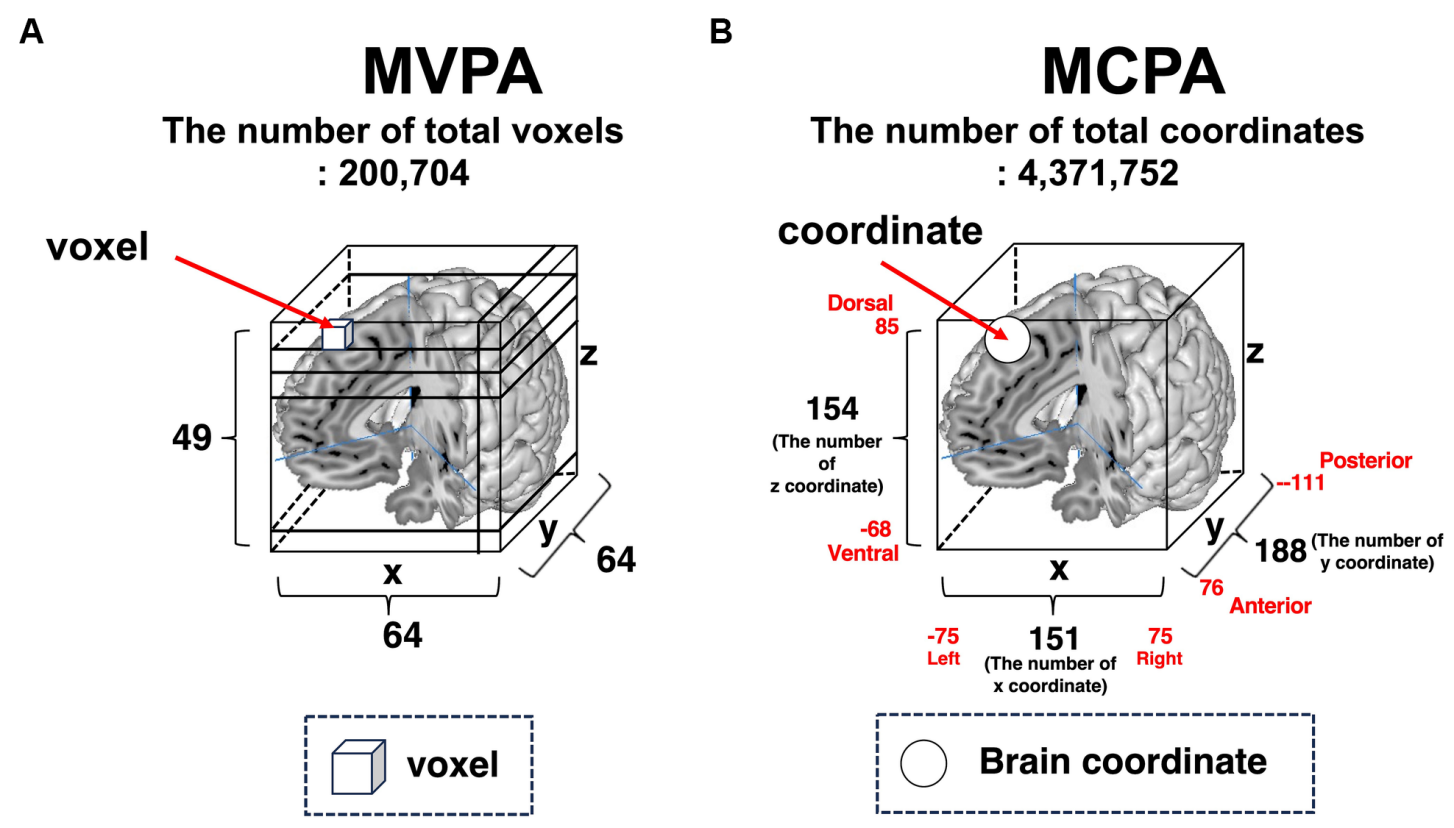
Basic brain information for conducting machine learning algorithms. **(A)** MVPA. There are 64 voxels in the *x*-axis direction, 64 voxels in the *y*-axis direction, and 49 voxels in the *z*-axis direction. The total number of voxels is 200,704 (64 × 64 × 49). **(B)** MCPA. Overall, 151 coordinates from left to right sides are lined in the *x*-axis direction, 188 coordinates from anterior to posterior are lined in the *y*-axis direction, and 154 coordinates are lined in the *z*-axis direction. The total number of coordinates is 4,371,752 (151 × 188 × 154). MVPA, multi-voxel pattern analysis; MCPA, multi-coordinate pattern analysis.

The information form of both the MVPA and MCPA is depicted in Figure 2. In MVPA (Figure 2A), voxel information is used as a feature variable. A voxel is a cube that contains information on the blood oxygen level-dependent (BOLD) signals obtained by functional magnetic resonance imaging (fMRI) experiences, which represents the presence or absence of activation. In MCPA (Figure 2B), coordinate information is used for analysis. The coordinate information mainly consists of activated foci in reported publications, although the deactivated voxels are included in the MVPA. Both feature variables are the same in terms of three-dimensional information regarding a brain space; however, the type of values and data formats used as feature variables are distinctive.

**Figure 3:**
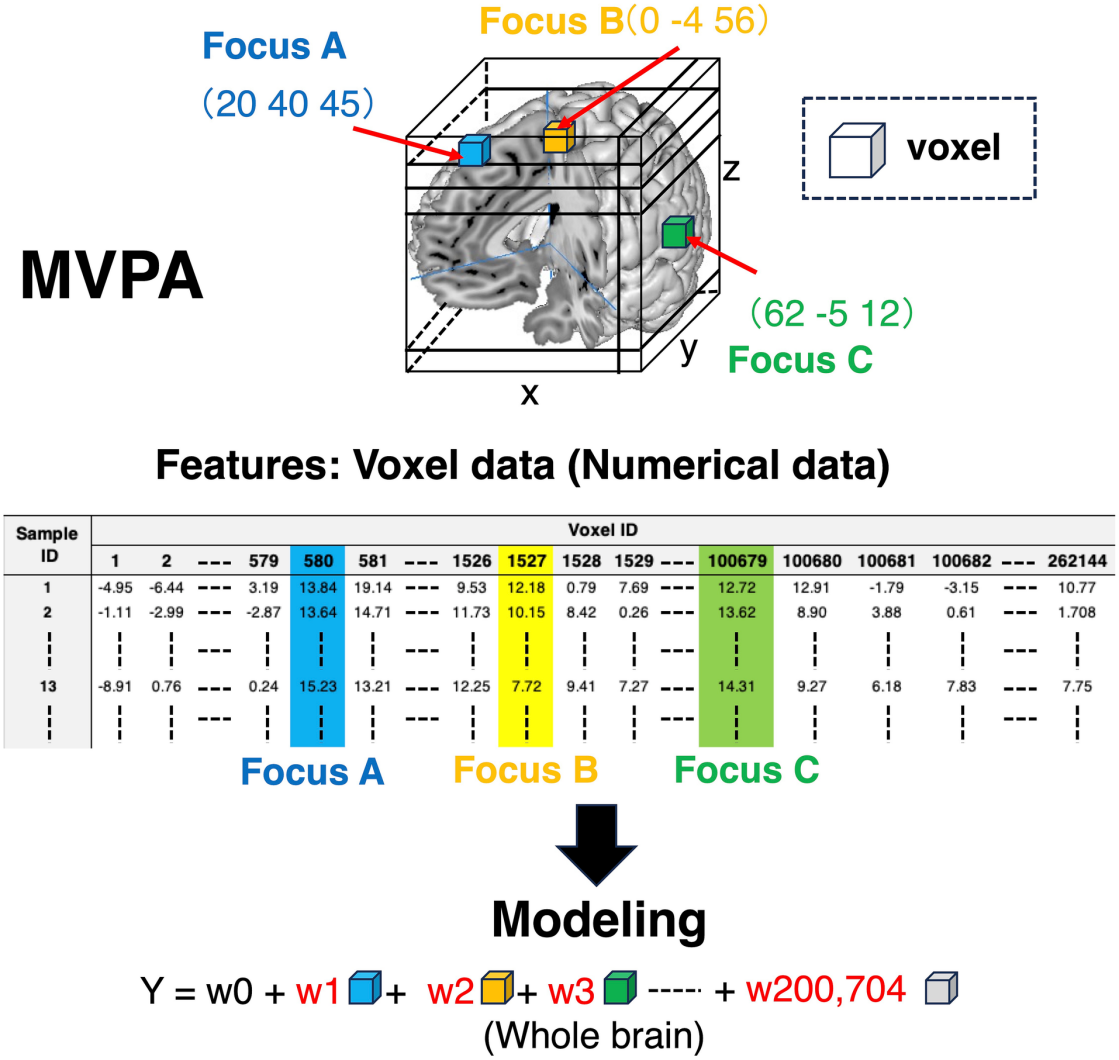
Feature variables for conducting machine learning algorithms in MVPA. Colored cubes represent voxels in the 3D brain picture. The information contained in these voxels is transformed into the matrix form to construct machine learning algorithms. As for the features in the matrix, the row in the matrix represents sample ID, and the column is voxel ID. The values in the matrix are voxel values, which represent BOLD signals. Thus, the voxel information is numerical values. BOLD, blood oxygen level-dependent; MVPA, multi-voxel pattern analysis.

**Figure 4:**
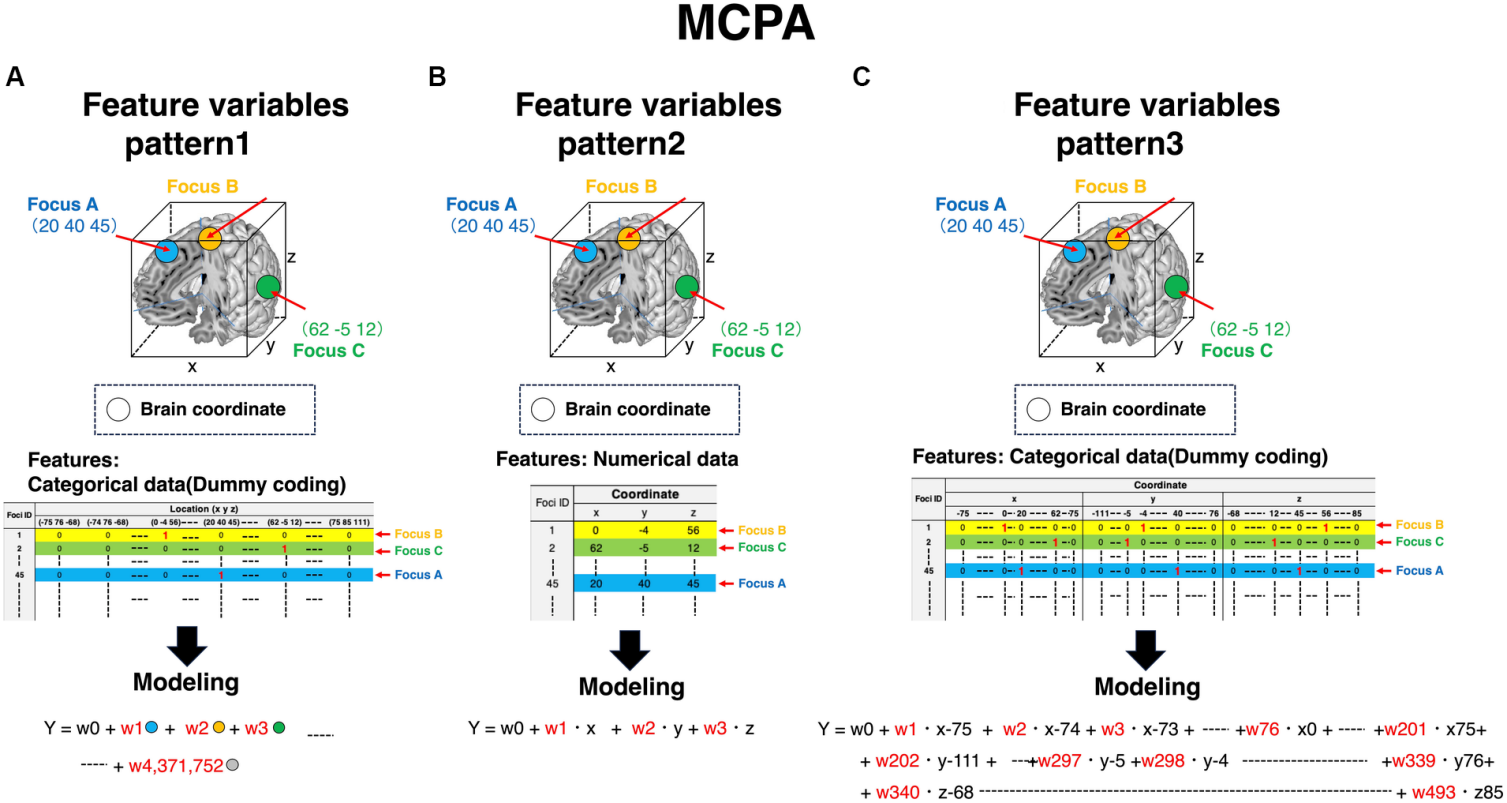
Feature variables for constructing machine learning algorithms in MCPA. The row in all patterns represents foci ID. Feature variables are stored in the column, and the expression of the column is distinct in each pattern. The way of modeling is distinct depending on the patterns of the expressing feature variables. Accordingly, the obtained results are also distinct. **(A)** Feature variables of pattern 1 in MCPA. All feature variables are coded as dummy variables. The value “1” is given to only the activated locations. Accordingly, the value “0” is given to all elements in other columns. The number of the value “1” in each row is one piece. In modeling the machine learning algorithms, these binary-coded values in each column are adopted as feature variables. **(B)** Feature variables of pattern 2 in MCPA. The values of coordinates described in publications are directly adopted as feature variables in modeling the machine learning algorithms. **(C)** Feature variables of pattern 3 in MCPA. The value “1” is coded at only one element in each coordinate (x, y, z), corresponding to the activated brain coordinates. Accordingly, the number of the value “1” in each row is three pieces. These values are feature variables for modeling machine learning algorithms. MCPA, multi-coordinate pattern analysis.

Each voxel includes information on whether brain regions are activated or not (colored cubes in Figure 3, and colored columns in the feature variables corner in Figure 3). Each voxel includes the information of BOLD signals, which is a numerical variable. Given that the voxel information is used as a feature variable, we can specify what voxels contribute to the outcomes using machine learning models. Contrarily, as for MCPA, there are three patterns representing feature variables. First, pattern 1 (Figure 4A) is the natural extension of MVPA in terms of directly revealing activated locations. However, as the matrix, which is occupied by numerous zero values, becomes extremely sparse, calculating the data might be challenging. Second, pattern 2 (Figure 4B) is an approach for using the raw coordinate data in publications. As the dimension of the feature variables can be significantly reduced rather than the pattern (Figure 4A), and there is no requirement to perform any feature engineering, machine learning models can be easily constructed. However, although this pattern can reveal the contribution to outcomes in terms of the axis of brain coordinates, the contributing brain locations cannot be specified, i.e., the *y*-axis direction contributes to the outcomes because the weight of y is higher than that of the x and z coordinates. As for pattern 3 (Figure 4C), although the feature variables are a type of categorical data similar to the pattern (Figure 4A), the dimension of feature variables can be reduced to 1/10,000 of pattern 1 (Figure 4A), despite transforming the numerical coordinate data into the dummy data in each coordinate. The weights, calculated in each coordinate using machine learning models, enable the identification of brain locations that contribute to outcomes. Therefore, as pattern 3 (Figure 4C) is a promising approach, the present study adopted it.

**Figure 5:**
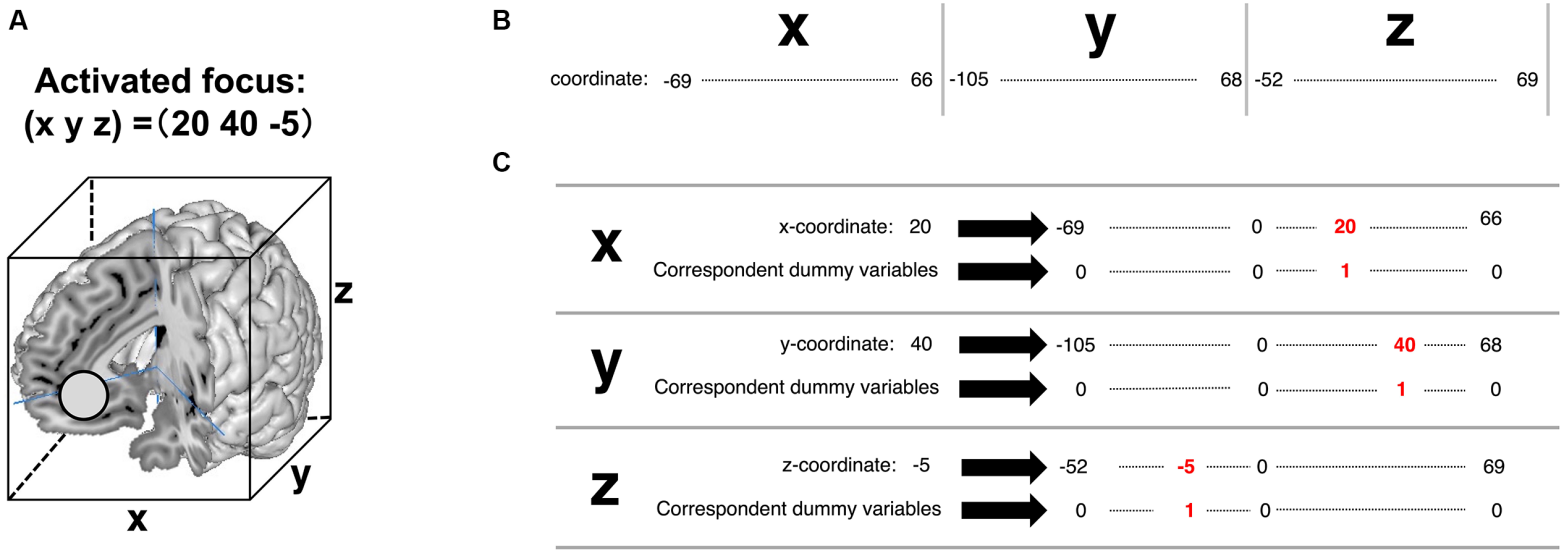
A detailed explanation of transforming activated focus to one-hot vectors in the feature variables of pattern 3. **(A)** Coordinate of the activated brain regions. **(B)** The column of the future variables. In the present study, the observed range in the x-coordinate was from −69 to 66. The observed range in the y-coordinate was from −105 to 68. The observed range in the z-coordinate was from −52 to 69. **(C)** The coded value “1” corresponds to the coordinates of the activated brain regions (written in red ink).

**Figure 6:**
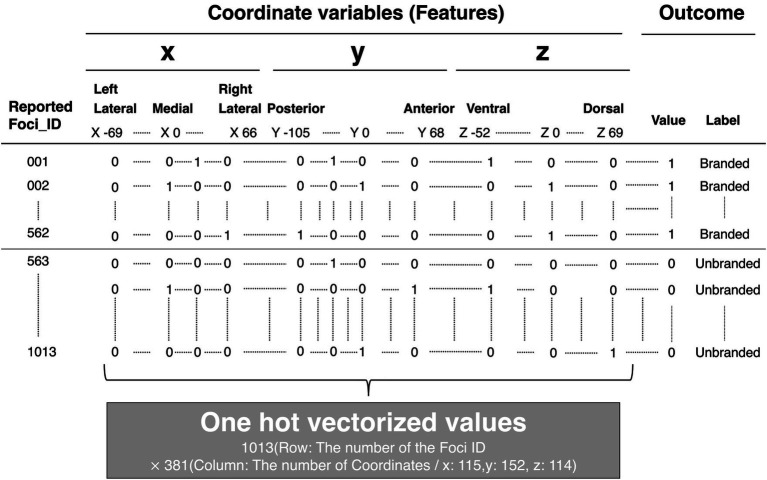
Structure of dataset for machine learning. The brain coordinates, one-hot vectorized, and outcomes, which represent the product choice, whether branded or not, were stored in columns. Each foci ID was stored in rows.

A detailed explanation of the feature engineering is provided in Figure 5. For example, Figure 5A is the coordinate of the activated brain regions. Each element in the coordinate was horizontally lined (Figure 5B). Subsequently, each element of the coordinate was transformed into dummy variables (Figure 5C). A detailed example is shown in Supplementary Table S2 (Excel file). Finally, these transformed coordinate data were organized into one data form (Figure 6). The x, y, and z coordinates totaled 115, 152, and 114 variables, respectively. The row is the foci ID, and the column contains coordinate information that was one-hot vectorized. Accordingly, these values in the columns were set as feature variables. We transformed all obtained coordinate data into one-hot vectors using scikit-learn 1.3.0 (Python module for machine learning).^3^

#### 2.2.2 Modeling algorithm

Although SVM is generally used as a machine learning algorithm for MVPA (Norman et al., 2006; Haxby, 2012), data after feature engineering are too sparse to apply SVM directly. Given that transforming sparse data into dense data has been confirmed to enhance the accuracy of machine learning approaches, we adopted the sparse partial least squares discriminant analysis (sPLS-DA) (Lê Cao et al., 2011) as a modeling algorithm. The sPLS-DA modifies the PLS-DA algorithm (Barker and Rayens, 2003; Boulesteix and Strimmer, 2007); the PLS-DA is a natural extension of the PLS algorithm (Boulesteix and Strimmer, 2007) that deals with categorical values as outcome variables.

First, the PLS-DA has latent components such as the PLS. *X* is a feature variable, and *Y* is an outcome variable constituting the dummy matrix. Here, latent components $t_{h}=X_{h}a_{h}\;$ and $u_{h}=Y_{h}b_{h}$ . *h*(1, 2 …, *H*) are the number of dimensions in the components. Both $a_{h}$ and $b_{h}$ are coefficients representing the importance of contributing to each component. Both $X_{h}$ and $Y_{h}$ are residual matrices. The PLS-DA is calculated by maximizing the following covariance formula (Eq. 5).


(5) $$\underset{\left(a_{h},b_{h}\right)}{\max}\begin{array}{c} \mathrm{cov}\left(X_{h}a_{h},Y_{h}b_{h}\right),s.t.{ah}_{2}={bh}_{2}=1\;\\ \; \end{array}$$

Thus, the feature variable *X* is decomposed into *H* components.

Second, the sPLS-DA is an approach combined with the L1 regularization method. The equation to maximize covariance formula is as follows (Eq. 6):


(6) $$\underset{\left(a_{h},b_{h}\right)}{\max}\begin{array}{c} \mathrm{cov}\left(X_{h}a_{h},Y_{h}b_{h}\right),s.t.{ah}_{2}={bh}_{2}=1\;and\;{ah}_{1}\leq \lambda_{h}\;\\ \; \end{array}$$

This modification enables the assessment of feature variables. $\lambda_{h}$ is the regularization parameter which controls the influence of the regularization term ${ah}_{1}$ .

Hence, the sPLS-DA approach has preferable characteristics for transforming sparse data into dense data using a decomposition method and assessing the contributing feature variables to outcome variables by L1 regularization. Although the convolutional neural network (CNN)-based classification model may be useful as an alternative approach, it cannot clarify the contribution of feature variables to the outcome. Therefore, even though the data may have an extremely sparse structure and high-dimensional feature variables, the sPLS-DA can be expected to address this issue.

**Figure 7:**
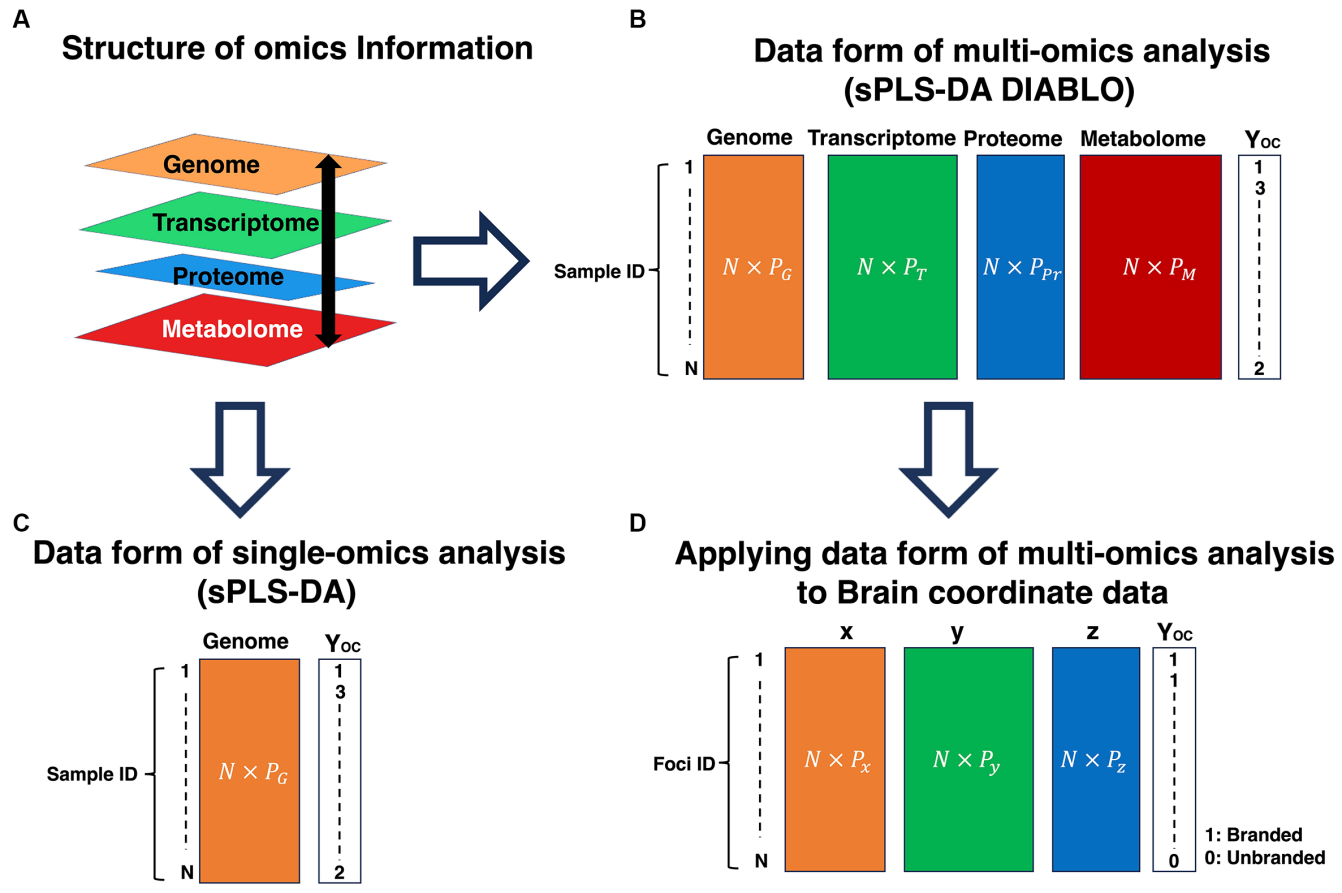
Explanation of omics information analysis and application of the multi-omics analysis data form to brain coordinates data. **(A)** Structure of omics information. **(B)** Data form of the multi-omics analysis (sPLS-DA DIABLO). **(C)** Data form of single-omics analysis (sPLS-DA). **(D)** Applying data form of multi-omics analysis to the brain coordinate data. P represents variables. The different number of variables in each omics information is allowed. However, the same number of N should be required among omics information. OC is a categorical variable. OC, outcome; G, Genome; T, Transcriptome; Pr, Proteome; M, Metabolome. sPLS-DA, sparse partial least squares discriminant analysis; DIABLO, data integration analysis for biomarker discovery using latent components.

Third, the data integration analysis for biomarker discovery using the latent variable approaches for omics studies (DIABLO) method is an extension of the sPLS-DA, which is an analysis algorithm for single omics, to analyze multi-omics data by integrating each omics (Singh et al., 2019). The overview of omics information analysis is depicted in Figure 7. Omics information is biological information, such as genomics, transcriptomics, proteomics, metabolomics, and lipidomics (Figure 7A). As shown in Figure 7A, although this information has distinctive roles, the information hierarchically restricts and has relationships with each other. Multi-omics analysis is an analysis to reveal the interaction of each omics. In the multi-omics analysis, as each omics information is dealt with in a different mode, this omics information is not calculated in a mixed-up manner (Figure 7B), unlike single-omics analysis (Figure 7C). Similar to the omics, the brain coordinates (x, y, z) represent activated locations in brain space and have relationships with each other. Therefore, as each coordinate can be distinctively dealt with, calculating a coordinate in a mix-up manner might be inappropriate. Unlike the sPLS-DA, the DIABLO method can treat each coordinate as a mode; thus, we adopted the DIABLO method to treat each coordinate as a block (Figure 7D). A brief explanation of the DIABLO method in PLS-DA is as follows:


(7) $$\underset{a_{h}^{\left(1\right)},\cdots a_{h}^{\left(Q\right)}}{\max}\begin{array}{c} \overset{Q}{\underset{q,j=1,q\neq j}{\sum }}c_{q,j}\mathrm{cov}\left(\begin{array}{l} X_{h}^{\left(q\right)}a_{h}^{\left(q\right)},\\ X_{h}^{\left(j\right)}a_{h}^{\left(j\right)} \end{array}\right),s.t.a_{h}^{\left(q\right)}2=1\;and\;a_{h}^{\left(q\right)}1\leq \lambda^{\left(q\right)}\;\\ \; \end{array}$$

where Q denotes the feature group data sets $X^{\left(1\right)}\left(N\times P_{1}\right),X^{\left(2\right)}\left(N\times P_{2}\right),\cdots X^{\left(Q\right)}\left(N\times P_{Q}\right)$ . *q* = 1,2,⋯ *Q*. In this study, *Q* expresses the number of coordinate elements (“x,” “y,” “z”) as blocks. P is feature variables (each coordinate variable; x10, x20, y10, y5, z10, z60, etc.…), *N* is the number of sample, $c_{q,j}$ is the design matrix, $X_{h}^{q}$ is the deflated residual matrix of the data set $X^{q}$ , *h* is the number of components, $a_{h}^{q}$ is the loading vector on the component *h*. $\lambda^{q}$ is the regulation parameter (L1 regulation). When executing discriminant analysis, $X^{q}$ is replaced with the outcome dummy matrix *Y*.

We conducted sPLS-DA using R and mixOmics (R packages).

## 3 Results

Both the ALE and MCPA analysis provided individual statistical results: ALE values were calculated using an ALE algorithm, and loading values, which is coefficients for the contribution of coordinates to outcomes, were calculated using sPLS-DA DIABLO. The detailed explanations are described below.

### 3.1 ALE

**Figure 8:**
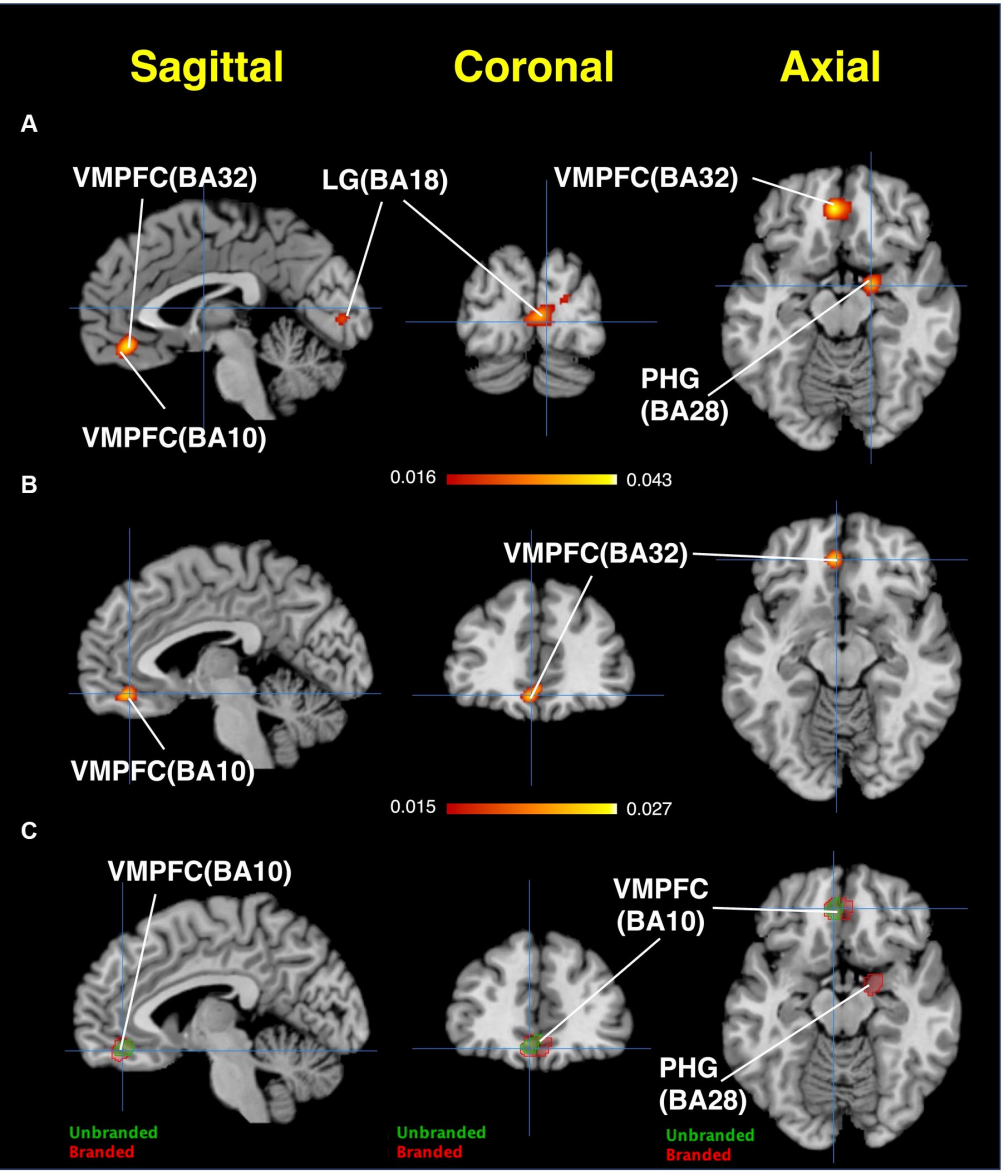
Results of ALE: Activated brain regions. **(A)** Branded foods-related decision-making. **(B)** Unbranded foods-related decision-making. **(C)** The region of interests (ROIs) map overlayed by both branded and unbranded foods-related decision-making. Red areas represent brain regions related to branded foods-related decision-making. Green areas represent brain regions related to unbranded foods-related decision-making.

The ALE results are presented in Figure 8 and Table 1. Regarding branded-food-related brain regions, activation was observed in the lingual gyrus, cuneus, VMPFC, and parahippocampal gyrus (BA28, close to the amygdala) (Figure 8A). The brain region activated by unbranded foods is the VMPFC (Figure 8B). Overlapping brain regions between branded and unbranded food were observed in the VMPFC (Figure 8C).

**Table 1:** Results of ALE.

Cluster #	Side	Brain region	BA	Peak voxel coordinates (MNI)			ALE values	Cluster size (mm^3^)
				x	y	z		
Branded foods								
1	R	Lingual Gyrus	BA18	6	−88	4	0.0349	6,368
	R	Lingual Gyrus	BA18	8	−74	−2	0.0319	
	R	Cuneus	BA17	22	−86	16	0.0205	
2	L	Anterior Cingulate (VMPFC)	BA32	−4	40	−8	0.0434	2,296
3	R	Parahippocampal Gyrus	BA28	18	−4	−16	0.0354	1,336
4	L	Lingual Gyrus	BA18	−18	−74	−4	0.0279	1,032
Unbranded foods								
1	L	Medial Frontal Gyrus (VMPFC)	BA10	−6	42	−16	0.0250	1,064

ALE, activation likelihood estimation; BA, Brodmann areas; MNI, Montreal Neurological Institute; VMPFC, ventral medial prefrontal cortex.

### 3.2 MCPA

**Figure 9:**
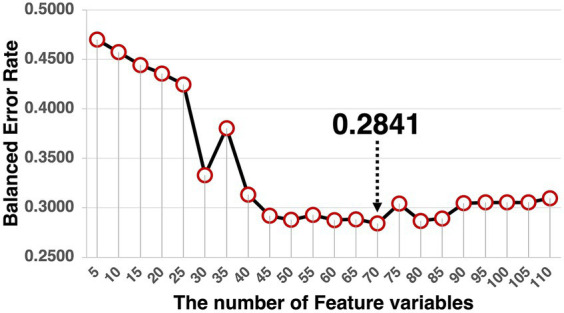
The trajectory of the balanced error rate. The lowest balanced error rate was 0.2841 at 70 feature variables.

The model was validated by splitting the dataset into two sets of training data (*n* = 831) and test data (*n* = 200). Because the variance of the feature variables was too small to conduct a cross-validation method determining the optimal number of variables, this number was determined by calculating the balanced error rate. The balanced error rate is the performance index for the learned model assessed by the test dataset, based on the number of feature variables from 5 to 110 (Figure 9). Given that 70 feature variables were the minimum value of the balanced error rate (0.2841), we selected 70 effective feature variables for each component. The balanced error rate of 0.2841 was above the chance level. The confusion matrix is listed in Table 2.

**Table 2:** Confusion matrix.

		Predicted	
		Branded foods	Unbranded foods
Actual	Branded foods	90	27
	Unbranded foods	28	55

**Figure 10:**
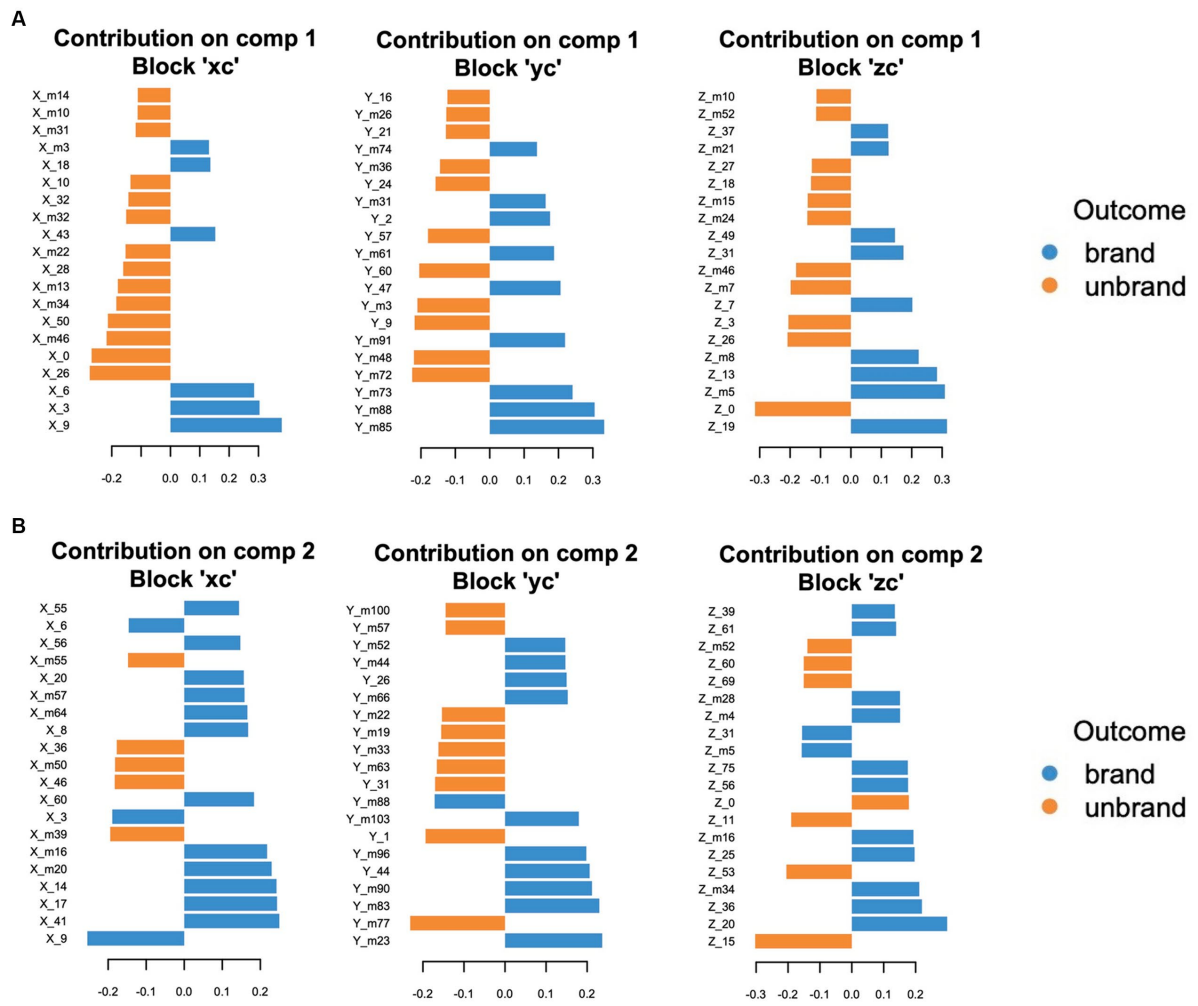
The top 20 ranked loading values in each coordinate. **(A)** Component 1. **(B)** Component 2. Blue bars represent contributed variables to branded foods-related decision-making. Orange bars represent contributed variables to unbranded foods-related decision-making.

The loading values of each component, which are indices that contribute to the discrimination between branded and unbranded foods, are shown in Figure 10 and Supplementary Table S3. The loading values corresponded with $a_{h}^{q}$ in Eq. 7. The loading values are weights on components (latent variables) and are calculated on each component. The loading values represent the contribution to the outcome variables. We conducted the sPLS-DA DIABLO algorithm by setting two components. Unlike PLS, the discriminant performances are more crucial than the explained variance in the PLS algorithm family. Nevertheless, explained variances can be referable by considering the contribution to the quantitative measurements for explaining the dominating components in the data. Each explained variance of coordinates is described as follows: x-coordinate: component 1 = 0.00880, component 2 = 0.00876; y-coordinate: component 1 = 0.00662, component 2 = 0.00661; and z-coordinate: component 1 = 0.00662, component 2 = 0.00661. The detailed results are described below. Although the variances of both components in each coordinate were almost similar, explained variances of component 1 in each coordinate yielded slightly higher values.

X_9 in component 1 was the most effective variable contributing to the prediction of consumer decision-making related to branded food. Subsequently, X_3 and X_6 strongly influence branded food-related consumer decision-making. Similar to the top-tier variable group, the coordinates belonging to the second group were also positioned in the relatively medial regions (X_m3, X_18), except X_43. The x coordinates with high loading values in unbranded foods were mainly placed on the lateral side of the brain in both components 1 and 2, although the medial part of the coordinates (X_0, X_10) was partly observed in component 1 (component 1: X_26, X_m46, and X_50; component 2: X_39, X_46, and X_m50). Regarding the y-coordinates in branded foods, the coordinates of the posterior sides were dominant in components 1 and 2 (component 1: Y_m85, Y_m88, and Ym_73; component 2: Y_m83, Y_m90, Y_m96, and Y_m103). Regarding the results for unbranded foods, the coordinate variables in the anterior and posterior parts of the brain regions contributed to component 1 (Y_m72, Y_m48, Y_9, Y_m3, and Y_60), whereas the coordinate variables from the anterior to posterior parts of the brain contributed to component 2 (Y_m77, Y_1, Y_31, Y_m63, and Y_m33). The z-coordinates with high loading values in branded foods were organized around the middle area of the brain on the sagittal plane in both components 1 (Component 1; Z_19, Z_m5, Z_13, Z_m8, and Z_7). Regarding component 2 in branded foods, the z-coordinates were broadly scattered from the ventral to the dorsal regions of the brain (component 2; Z_20, Z36, Z_m34, Z_25, and Z_m16). Regarding the z coordinates of unbranded foods, there were no convergent brain areas with high loading values in components 1 and 2 (component 1; Z_0, Z_26, Z_3, Z_m7, and Z_m46/component 2; Z_15, Z_53, Z_11, Z_0, and Z_69). Thus, the medial and posterior regions contribute to discriminating branded foods, whereas the lateral regions play a role in identifying unbranded foods.

**Figure 11:**
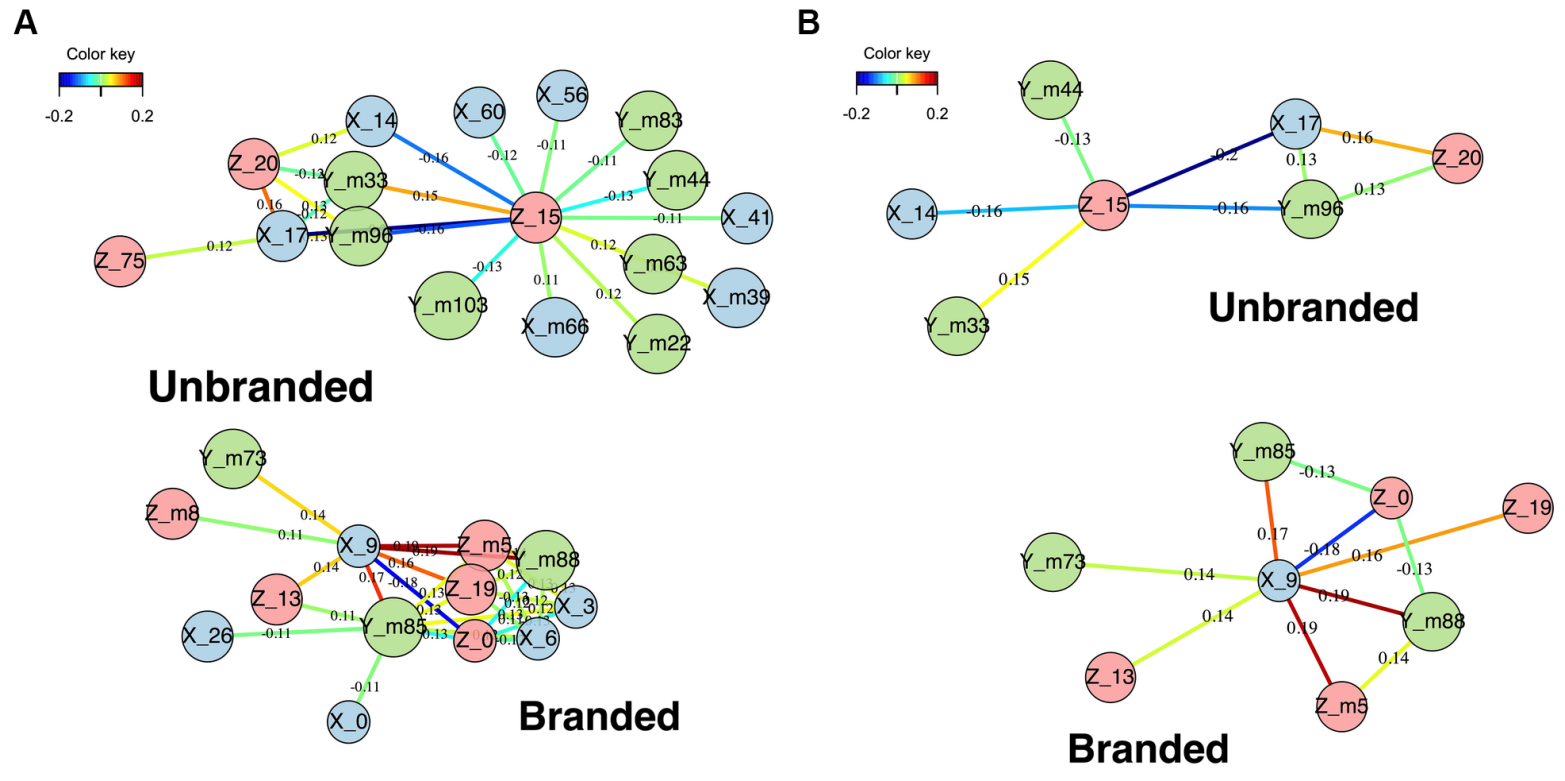
Relevance network between coordinates. **(A)** Relevance networks with a cut-off rate of 0.11. **(B)** Relevance networks with a cut-off rate of 0.13. The edge colors of both yellow and red represent strong ties. Light blue and blue edge colors represent weak ties. Green edge color represents relatively strong ties.

Because network analysis can visualize the correlation structure between coordinate variables, a connection pattern among an element of coordinates, which is the spatial position of the brain, might be innately reconstructed. The structure of the association between the feature variables was analyzed using the relevance network approach implemented in the mixOmics package (Figure 11). The edges of the network, which are calculated by the integrated method between canonical correlation analysis and sPLS, represent a connection strength between the different elements of the coordinate, which are referred to as “nodes” (González et al., 2012). In Figure 11, the circle is a node, and the line represents an edge. Values on the edges are referred to as the association score, which is calculated as follows:

Here, X(n×p) and Y(n×q) are two block data matrices. X is a feature variable, whereas Y is an outcome variable. *n* is the number of samples, and *p* and *q* are the number of variables. $X^{p}$ denotes *p*th variable in the X block data ( j=1⋯,p) . $X^{q}$ denotes *q*th variable in the Y block data ( k=1⋯,q) . $M_{j}^{k}$ represents an association score of the *j*th row and *k*th column of the *M*. X (Eq. 8) and Y (Eq. 9) are decomposed using PLS algorithm as follows:


(8) $$X=U^{1}{\left(\phi^{1}\right)}^{\prime }+U^{2}{\left(\phi^{2}\right)}^{\prime }+\cdots +U^{r}{\left(\phi^{r}\right)}^{\prime }$$

(9) $$Y=U^{1}{\left(\varphi^{1}\right)}^{\prime }+U^{2}{\left(\varphi^{2}\right)}^{\prime }+\cdots +U^{r}{\left(\varphi^{r}\right)}^{\prime }+E^{r}$$

$U^{l}$ is the latent variables ( l=1⋯,r ). *r* represents the number of the decomposed dimensions. Here, $U^{l}=Xa^{l}$ . $a^{l}$ is a loading value, and $\phi^{l}$ and $\varphi^{l}$ are coefficients on $U^{l}$ in regression. $E^{r}$ is the residual matrix ( l=1⋯,r ). $u_{l}$ denotes the standard deviation of $U^{l}$ . By using the orthogonal properties of the latent variables and decompositions in (Eq. 8) and (Eq. 9), $x_{l}^{j}$ = correlation ( $X^{j},U^{l}$ ) = $u_{l}\phi_{j}^{l}$ and $y_{l}^{k}$ = correlation ( $Y^{k},U^{l}$ ) = $u_{l}\varphi_{k}^{l}$ . Thus, the association score $M_{k}^{j}$ is defined as (Eq. 10):


(10) $$M_{k}^{j}=x^{j},y^{k}=\overset{d}{\underset{l=1}{\sum }}x_{l}^{j}y_{l}^{k}=\overset{d}{\underset{l=1}{\sum }}u_{l}^{2}\phi_{j}^{l}\varphi_{k}^{l}\approx correlation\left[\begin{array}{l} X^{j},\\ Y^{k} \end{array}\right]\;\left(d<r\right)$$

Therefore, the association score $M_{k}^{j}$ enables the measurement of the relationship of feature variables across blocks.

As shown in Figure 11A, nodes of the lateral, posterior, and dorsal sides were assessed in the network of unbranded food choice behavior. Regarding the network of branded food choice behavior, the medial side, posterior side, and middle of the vertical line of the brain axis were characteristically observed. The network shown in Figure 11B is sharpened and more clearly structured than that in Figure 11A, owing to the application of a higher cut-off rate (a thresholded association score for visualization). Therefore, we mainly analyzed the network structure based on Figure 11B. The branded food-related network represents strong connections between the node corresponding with the right medial region (X9), left posterior region (Ym_88), and center areas of z (Z_m5). Considering the exclusion of negative connected nodes and given that these nodes were connected in a triangle, these connected coordinates correspond with the following foci: (9–88 –5). This coordinate corresponds to the lingual gyri. This result is consistent with that of the ALE. Regarding the unbranded food-related network, the connection groups are the right lateral region (X_17), the posterior region (Ym_96), and the dorsal region (Z_20). These positively connected nodes correspond with brain foci (17–96 20), which is the cuneus (BA18). For brain regions related to unbranded foods, the dorsal and lateral sides of the brain regions were characteristically detected, although these areas were inconsistent with the ALE results.

## 4 Discussion

In this study, using ALE and MCPA, we identified three characteristic brain regions related to branded foods: the lingual gyrus, PHG, and VMPFC. As the lingual gyrus was validated by the results of both ALE and MCPA, this might be the most robust and unique brain region related to consumers’ branded food decision-making. Although MCPA did not detect the PHG or VMPFC, these brain areas might also play a crucial role in mental processes related to branded food decision-making. All of these regions are located in the medial part of the brain and match the cortical midline structure (CMS) (Northoff and Bermpohl, 2004). Furthermore, the CMS is associated with self-referential processing (Northoff and Bermpohl, 2004), suggesting that the mental processes of consumer decision-making regarding branded foods may underlie this process. According to several marketing studies, brand equity must, most importantly, allow consumers the possibility of self-expression (Aaker, 1996; Fournier, 1998). To achieve this benefit, tied information between the brands and consumers is required. Since the benefit is a self-referential process, our results are consistent with the previous marketing literature (Aaker, 1996; Fournier, 1998). Regarding brain regions related to unbranded foods, only the VMPFC was characteristically activated. Although the lateral frontal region was activated, no specific converged brain regions were detected. The VMPFC had overlapping brain regions between branded and unbranded food-related decision-making. This suggests that the VMPFC might be the core brain region in food-related consumer decision-making, regardless of whether branded foods are used as experimental stimuli. This region is almost consistent with that identified by McClure et al. (2004) in famous study using blindfolded brand logos, although there are slight differences in that the decision-making area in the present study was located in the left side of the ventral PFC, whereas that in McCure et al.’s study was located in the right side of the ventral PFC. The VMPFC is well known as the value calculator and integrator in the brain (Delgado et al., 2016). This region plays a crucial role in subjective reward processing and subjective value decision-making, including social aspects (Bartra et al., 2013; Sescousse et al., 2013; Clithero and Rangel, 2014). Further, it evaluates objects in terms of pleasantness and unpleasantness (Peters and Büchel, 2010; Yin et al., 2021). Yin et al. (2021) demonstrated that the VMPFC facilitates the prioritization of self-related stimuli in cooperation with brain regions comprising a working memory network. Thus, this region may be associated with assessing the value of objects and matters regarding self-relevancy. Since activation of this brain region overlapped between branded and unbranded foods in this study, mental processes related to self-related valuation might play a crucial role in food-related decision-making, regardless of whether the food is branded.

The lingual gyrus is associated with episodic and autobiographical memories related to visual information (Burianova and Grady, 2007; Burianova et al., 2010). The visual imagination plays a crucial role in the retrieval of autobiographical memories (Greenberg and Knowlton, 2014). de Gelder et al. (2015) observed activation of the lingual gyrus in blind individuals with bilateral primary visual cortex lesions in an auditory and visual imaginary task. When reading sentences, the lingual gyrus was activated regardless of the participants’ previous information (Mo et al., 2006). Jin et al. (2009) suggested that the lingual gyrus engages in predictive inferences in terms of contextual comprehension by referring to long-term memory. This suggests that the lingual gyrus is associated with the visual construction of spatial scenes. Moreover, this region engages in creative thinking (Zhang et al., 2014, 2016; Jauk et al., 2015). Considering that divergent thinking is a type of creativity that explores multiple options from various perspectives, it requires many cognitive resources (Guilford, 1967; Runco and Acar, 2019). Given that the lingual gyrus is associated with vivid visual memory, visual imagery contributes to solving complex problems while executing divergent thinking (Zhang et al., 2016). Moreover, the lingual gyrus, in cooperation with other cortical regions, may play a crucial role in semantically assembling and assimilating vividly visualized representations stored in the long-term memory system (Zhang et al., 2014). Visual imagery of the lingual gyrus may lead to ideational originality and fluency (Jauk et al., 2015). Thus, this region may mentally operate and integrate visual elements, including spatial information, in cooperation with other brain regions.

The PHG (BA28) engages in memory-related mental processes, such as episodic memory, autobiographical memory, associative memory, encoding, and recognition (Boccia et al., 2019). Given that the PHG (BA28) is closely positioned to the amygdala, these connected brain regions are involved in emotional memory processing, regardless of input sensory modalities, such as visual, auditory, and odor (Kesner and Rogers, 2004; Buchanan et al., 2006; Dahmani et al., 2018; Zhou et al., 2021). Memory processing driven by PHG (BA28) is involved in spatial information and navigation (Ekstrom and Bookheimer, 2007; Epstein, 2008). These features can lead to the formation of contextually associative memory processing and navigational functions (Aminoff et al., 2013). Interestingly, the connection between the lingula gyrus and PHG (BA28) is associated with predictive inference (Jin et al., 2009). When reading sentences, predictive inferences facilitate understanding beyond the actual content by taking contextual information (Allbritton, 2004). Contextual associative memories based on visual imagery, which are derived from the connection between the PHG and the lingual gyrus, might contribute to predictive inferences.

Moreover, both the PHG, including BA28, and VMPFC are the major regions composed of the medial temporal lobule subsystem (MTL subsystem), which is a sub-system of the default mode network (Andrews-Hanna, 2012; Ward et al., 2014). The MTL subsystem is associated with imagining the future self and contextually reconstructs autobiographical and episodic memories (Addis et al., 2009; Andrews-Hanna et al., 2010b), a process spontaneously driven by imagery (Andrews-Hanna et al., 2010a). The MTL subsystem is involved in spontaneous subjective memory processing. Given that a connection between regions of the MTL system and the lingual gyrus has been observed in resting-state functional connectivity studies (Ward et al., 2014; Lee and Xue, 2018), the lingual gyrus may be involved in mental processes derived from the MTL subsystem.

The novelty of the present study lies in the identification of a unique and converged brain region for branded food-related decision-making using both the ALE and MCPA, regardless of experimental task differences, and provides MCPA, which is a new approach for detecting brain regions using the sPLS-DA DIABLO method, based on the obtained coordinate data. To the best of our knowledge, this is the first study to predict mental processes and behaviors using published coordinate data. This proposed approach, namely the MCPA, might extend the usage of CBMA and help researchers provide additional insights into CBMA results. The MCPA using sPLS-DA DIABLO takes advantage of reducing dimensions compared with that using direct brain location data (Figure 4A: MCPA pattern 1) as feature variables and for identifying brain locations, unlike raw coordinate data (Figure 4B: MCPA pattern 2). However, this study has several limitations; although our findings may have comprehensive validity in the food category, several factors remain to be addressed. Because analyses were conducted concerning brain regions by focusing on a single category, concerns about fluctuating activated brain regions could be excluded depending on the categories. However, the present study did not consider the influence of the differentiation of demographic variables (sex and age) or psychological variables (sense of values and personality). These variables are crucial in market segmentation strategies and may be crucial to the perceptions of and benefits of a brand. The present study did not consider the quantity and quality of consumers’ brand knowledge, including the relationship between brands and consumers. Even if consumers perceive the same brand, those who are favorable to the brand may have more positive attitudes (Batra et al., 2012). Additionally, there are some concerns in CBMA. According to Salimi-Khorshidi et al. (2009), the IBMA approach is the most optimized and ideal method for analyzing activated brain regions because of its ability to use massive amounts of information, including the absence of activated brain regions. When applying and interpreting the present study, it should be recognized that the results depend on the limited information. Another concern regarding the CBMA is publication bias that is implicitly and potentially underlain in meta-analytical results, including this study (Rothstein et al., 2005). Activated foci, which depend on a small number of studies in each cluster, might have publication bias. Therefore, the results of this study should be interpreted cautiously. Although the MCPA approach using the sPLS-DA DIABLO worked very well in the present study, the data of the present study had a very low variance for performing cross-validation. We should further refine the MCPA for addressing data with low variance in some way when conducting cross-validation. Another concern regarding the MCPA is addressing the imbalanced data. The present study had 562 and 469 samples of branded and unbranded food choice behavior, respectively. The training dataset was divided into the data of 445 branded food choice behaviors and 386 non-branded food choice behaviors. The testing dataset was divided into the data of 117 branded and 83 non-branded food choice behaviors. Although the total, training, and testing datasets had almost similar sample sizes, slightly imbalanced data was observed in both datasets. Imbalanced data cause false positive problems, that is, a predictor wrongly predicts a negative class as a positive class (He and Garcia, 2009). This problem tends to be generated in minority-class data. To address this problem, several data augmentation methods, such as SMOTE (Chawla et al., 2002) and deep generative models (Xu et al., 2019), are promising methods. These methods might process imbalanced data into balanced data. Conducting the machine learning algorithm based on the imbalanced data might cause inconsistent results between the ALE and MCPA in the unbranded food-related brain regions. Thus, further research is needed to clearly identify branded food-related brain regions.

## 5 Conclusion

Our results indicate that the lingual gyrus might be the primary discriminative brain region for branded and unbranded food-related decision-making. Subjective contextual associative memories driven by the connected brain regions between the PHG and lingual gyrus are likely to form characteristic mental processes in branded food-related decision-making. Because these processes are operated by mental imagery, marketers should plan and execute a brand strategy that aims to enable consumers to spontaneously drive self-relevant memory resources. Thus, the aim is for consumers to inwardly imagine future scenes consuming the brand, and subsequently associate it with pleasantness.

## Data availability statement

The raw data supporting the conclusions of this article will be made available by the authors, without undue reservation.

## Author contributions

SW: Conceptualization, Data curation, Formal analysis, Funding acquisition, Investigation, Methodology, Project administration, Resources, Software, Supervision, Validation, Visualization, Writing – original draft, Writing – review & editing.

## Footnotes

^1^http://www.brainmap.org/, accessed on August 1, 2023.

^2^http://ric.uthscsa.edu/mango/, accessed on August 1, 2023.

^3^https://scikit-learn.org/stable/, accessed on August 1, 2023.
