# Supplementary material for: Identifying distinctive brain regions related to consumer choice behaviors on branded foods using activation likelihood estimation and machine learning
Source: Front Comput Neurosci. 2024 Jan 17;18:1310013. doi: 10.3389/fncom.2024.1310013 (PMC10875973; doi:10.3389/fncom.2024.1310013)
Supplement: Supplementary file 1 [file Table_1.DOCX]

Supplementary Material

Identifying distinctive brain regions related to consumer choice behaviors on branded foods using activation likelihood estimation and machine learning

**Table S1(a)**. **Branded** **foods studies included in the meta-analysis.**

| **Experiment** | **Subject** | **Foci** | **Experiment stimuli** | **Detailed information** |
| --- | --- | --- | --- | --- |
| McClure et al.(2004) | 16 | 7 | Brand logos/Carbonated drinks(Coke/Pepsi) | Culturally Familiar Drinks |
| Deppe et al.(2005b) | 22 | 16 | Package images with brand logos | Beer/Coffee |
|  |  | 2 |  |  |
|  |  | 8 |  |  |
|  |  | 11 |  |  |
| Koeneke et al.(2008) | 19 | 28 | Real products | Chochorate bars |
| Plassmann et al.(2008) | 20 | 8 | Wine | Intake |
|  |  | 10 |  |  |
|  |  | 12 |  |  |
|  |  | 6 |  |  |
|  |  | 2 |  |  |
|  |  | 5 |  |  |
|  |  | 8 |  |  |
|  |  | 1 |  |  |
| Kato et al.(2009) | 40 | 18 | Advertising with brand logos | Coke Ad/Pepsi Ad |
|  |  | 8 |  |  |
|  |  | 3 |  |  |
|  |  | 26 |  |  |
| Schaefer et al.(2011) | 12 | 2 | Package images with brand logos | Chochorates |
| Grabenhorst et al.(2013) | 13 | 2 | Fooods with Taste label/Health label |  |
|  |  | 4 |  |  |
|  |  | 2 |  |  |
|  |  | 2 |  |  |
|  |  | 1 |  |  |
|  |  | 3 |  |  |
|  |  | 4 |  |  |
| Bruce et al.(2014) | 17 | 7 | Brand logos | foods(60):pizzahut,kfc,etc./non foods(60):lego,spongebob,windows,etc. |
|  |  | 5 |  |  |
|  |  | 4 |  |  |
| Burger and Stice(2014) | 9 | 10 | Product with logo | Coke Ad/Non food Ad |
|  | 25 | 12 | Product & logo ad |  |
|  |  | 37 |  |  |
|  |  | 8 |  |  |
|  |  | 11 |  |  |
|  |  | 4 |  |  |
| Enax et al.(2015) | 40 | 9 | Food images with FT certified marks | Various food categories (chocolate, coffee, rice, etc..) |
|  |  | 5 |  |  |
|  |  | 7 |  |  |
|  |  | 10 |  |  |
|  |  | 4 |  |  |
|  |  | 4 |  |  |
|  |  | 6 |  |  |
|  |  | 3 |  |  |
|  |  | 2 |  |  |
| Jung et al.(2018) | 34 | 21 | Food images with logo (social/conventional enterprises) | Confectionery(i.e., cookies, chocolate, bread, and Korean traditional rice cake) |
|  |  | 15 |  |  |
|  |  | 9 |  |  |
|  |  | 8 |  |  |
|  |  | 11 |  |  |
|  |  | 4 |  |  |
|  |  | 4 |  |  |
|  |  | 25 |  |  |
|  |  | 1 |  |  |
|  |  | 21 |  |  |
| Goedegebure et al.(2022) | 30 | 1 | Foods package images with brand logos | 180 food products were presented under the 3 conditions (normal., social, and quality focus) |
|  |  | 4 |  |  |
|  |  | 3 |  |  |
|  |  | 6 |  |  |
|  |  | 2 |  |  |
|  |  | 19 |  |  |
|  |  | 5 |  |  |
|  |  | 11 |  |  |
|  |  | 13 |  |  |
|  |  | 3 |  |  |
|  |  | 5 |  |  |
|  |  | 5 |  |  |
|  |  | 5 |  |  |
|  |  | 2 |  |  |
|  |  | 12 |  |  |

Abbreviations; FT, fairtrade

**Table S1(b)**. **Unbranded** **foods studies included in the meta-analysis.**

| **Experiment** | **Subject** | **Foci** | **Experiment stimuli** | **Detailed information** |
| --- | --- | --- | --- | --- |
| McClure et al.(2004) | 15 | 2 | Carbonated drinks(No branded) | Intake |
| O'Doherty et al.(2006) | 13 | 6 | Flavored drinks | Intake |
| Bray et al. (2008) | 23 | 3 | Food (liquid drink) | Intake |
| Plassmann et al.(2008) | 20 | 1 | Wine | Intake |
|  |  | 10 |  |  |
|  |  | 2 |  |  |
| Chib et al.(2009) | 19 | 1 | money/trinkets/snacks |  |
|  |  | 1 |  |  |
|  |  | 1 |  |  |
|  |  | 1 |  |  |
| Van der Laan et al.(2012) | 20 | 8 | Food packages and products(images) |  |
|  |  | 2 |  |  |
|  |  | 10 |  |  |
| Kang and Camerer(2013) | 27 | 17 | Food images |  |
| Lee et al.(2013) | 23 | 3 | Food images |  |
| Jimura et al.(2013) | 43 | 7 | Food (liquid drink) | Intake |
|  |  | 5 |  |  |
|  |  | 2 |  |  |
|  |  | 4 |  |  |
| Burger and Stice.(2014) | 9 | 11 | Milkshake |  |
| He Q et al.(2014) | 30 | 5 | Food images | High-calorie food(chocolate bars, cookies, ice cream, and potato chips) /Low-calorie food(celery, broccoli, and carrots.) |
|  |  | 1 |  |  |
|  |  | 7 |  |  |
|  |  | 1 |  |  |
| Giuliani and Pfeifer(2015) | 60 | 16 | Food images | Energy density(ED) and low ED foods(e.g., chocolate, cookies, carrots, corn etc..) |
|  |  | 12 |  |  |
|  |  | 3 |  |  |
|  |  | 16 |  |  |
|  |  | 15 |  |  |
|  |  | 2 |  |  |
|  |  | 8 |  |  |
|  |  | 9 |  |  |
| Petit et al.(2016) | 22 | 7 | Food images | Various food categories(e.g., junk food, healthy snacks) |
|  |  | 2 |  |  |
|  |  | 5 |  |  |
|  |  | 6 |  |  |
|  |  | 17 |  |  |
|  |  | 4 |  |  |
| Stuke et al(2016) | 38 | 5 | Drink images | Alcohol dirnks/Non-alcohol drinks |
|  |  | 4 |  |  |
| Tapp et al.(2017) | 8 | 4 | Food images | Beef steaks |
|  |  | 7 |  |  |
|  |  | 7 |  |  |
|  |  | 5 |  |  |
|  |  | 8 |  |  |
| Hege et al.(2018) | 23 | 3 | Food images | Meals |
|  |  | 1 |  |  |
|  |  | 1 |  |  |
|  |  | 1 |  |  |
| Huijsmans et al.(2019) | 47 | 1 | Food images | 144 different supermarket food items |
|  |  | 1 |  |  |
| Masterson TD et al(2019) | 41 | 10 | Food images | Energy dense foods  (e.g., Low:vegetables and fruit/High: candy and ice-cream) |
|  |  | 6 |  |  |
|  |  | 4 |  |  |
| Muñoz-Leiva et al.(2019) | 24 | 7 | Food images | Meals(restaurant dishes) |
|  |  | 5 |  |  |
|  |  | 12 |  |  |
|  |  | 13 |  |  |
| Setton et al(2019) | 16 | 26 | Food images(package) | Snack items |
|  |  | 43 |  |  |
|  |  | 9 |  |  |
|  |  | 26 |  |  |
|  |  | 13 |  |  |
|  |  | 3 |  |  |
| Tijssen et al.(2019) | 34 | 3 | Package images | Daily drinks |
|  |  | 7 |  |  |
|  |  | 1 |  |  |

**Table S2**. **One-hot vector corresponded to (20 40 -5).** Please take a look at the other attached Excel file.

**Table S3**. **Loading values in each coordinate variable.**

| **X** | | **Y** | | **Z** | |
| --- | --- | --- | --- | --- | --- |
| **Cordinate** | **Loading values** | **Cordinate** | **Loading values** | **Cordinate** | **Loading values** |
| **Component 1** | | | | | |
| X_9 | 0.379 | Y_m85 | 0.333 | Z_19 | 0.316 |
| X_3 | 0.304 | Y_m88 | 0.305 | Z_m5 | 0.309 |
| X_6 | 0.285 | Y_m73 | 0.241 | Z_13 | 0.283 |
| X_43 | 0.153 | Y_m91 | 0.219 | Z_m8 | 0.223 |
| X_18 | 0.136 | Y_47 | 0.206 | Z_7 | 0.202 |
| X_m3 | 0.131 | Y_m61 | 0.187 | Z_31 | 0.173 |
| X_48 | 0.106 | Y_2 | 0.176 | Z_49 | 0.145 |
| X_m18 | 0.095 | Y_m31 | 0.163 | Z_m21 | 0.124 |
| X_56 | 0.087 | Y_m74 | 0.138 | Z_37 | 0.123 |
| X_63 | 0.086 | Y_m12 | 0.122 | Z_29 | 0.108 |
| X_49 | 0.083 | Y_m94 | 0.107 | Z_16 | 0.106 |
| X_m45 | 0.082 | Y_m80 | 0.097 | Z_8 | 0.105 |
| X_m42 | 0.082 | Y_m97 | 0.090 | Z_m14 | 0.104 |
| X_25 | 0.078 | Y_23 | 0.082 | Z_1 | 0.100 |
| X_m53 | 0.074 | Y_m24 | 0.079 | Z_34 | 0.082 |
| X_17 | 0.072 | Y_43 | 0.072 | Z_m19 | 0.077 |
| X_m69 | 0.072 | Y_m55 | 0.072 | Z_m11 | 0.074 |
| X_m41 | 0.068 | Y_m25 | 0.071 | Z_43 | 0.065 |
| X_30 | 0.068 | Y_m44 | 0.067 | Z_m27 | 0.065 |
| X_16 | 0.059 | Y_m13 | 0.055 | Z_m23 | 0.062 |
| X_60 | 0.055 | Y_40 | 0.054 | Z_m28 | 0.059 |
| X_11 | 0.054 | Y_m1 | 0.042 | Z_39 | 0.058 |
| X_14 | 0.043 | Y_m43 | 0.038 | Z_m38 | 0.055 |
| X_m7 | 0.042 | Y_m78 | 0.036 | Z_20 | 0.036 |
| X_m5 | 0.032 | Y_m96 | 0.034 | Z_75 | 0.022 |
| X_m11 | 0.032 | Y_38 | 0.019 | Z_40 | 0.022 |
| X_66 | 0.027 | Y_m83 | 0.017 | Z_m22 | 0.021 |
| X_55 | 0.025 | Y_68 | 0.007 | Z_57 | 0.019 |
| X_64 | 0.024 | Y_m9 | 0.005 | Z_m39 | 0.018 |
| X_m57 | 0.023 | Y_51 | 0.004 | Z_m16 | 0.016 |
| X_19 | 0.023 | Y_m40 | 0.001 | Z_m44 | 0.016 |
| X_41 | 0.022 | Y_m52 | 0.000 | Z_m29 | 0.015 |
| X_m49 | 0.022 | Y_31 | -0.001 | Z_m31 | 0.012 |
| X_m64 | 0.013 | Y_m34 | -0.002 | Z_56 | 0.009 |
| X_m60 | 0.004 | Y_m100 | -0.003 | Z_61 | 0.008 |
| X_m20 | 0.003 | Y_7 | -0.003 | Z_m12 | -0.016 |
| X_m15 | 0.002 | Y_1 | -0.003 | Z_46 | -0.016 |
| X_39 | -0.004 | Y_37 | -0.006 | Z_42 | -0.016 |
| X_m21 | -0.008 | Y_m18 | -0.008 | Z_60 | -0.024 |
| X_m28 | -0.015 | Y_m19 | -0.012 | Z_69 | -0.024 |
| X_2 | -0.034 | Y_15 | -0.019 | Z_48 | -0.025 |
| X_m55 | -0.039 | Y_m59 | -0.023 | Z_m40 | -0.027 |
| X_31 | -0.047 | Y_m22 | -0.026 | Z_65 | -0.033 |
| X_m19 | -0.047 | Y_49 | -0.027 | Z_67 | -0.036 |
| X_7 | -0.047 | Y_m15 | -0.033 | Z_74 | -0.041 |
| X_m27 | -0.051 | Y_56 | -0.045 | Z_23 | -0.042 |
| X_m66 | -0.052 | Y_55 | -0.046 | Z_50 | -0.045 |
| X_m39 | -0.055 | Y_m8 | -0.046 | Z_15 | -0.046 |
| X_m56 | -0.055 | Y_m75 | -0.047 | Z_44 | -0.051 |
| X_m48 | -0.061 | Y_42 | -0.065 | Z_21 | -0.058 |
| X_27 | -0.062 | Y_m63 | -0.067 | Z_9 | -0.067 |
| X_35 | -0.066 | Y_m57 | -0.069 | Z_m50 | -0.088 |
| X_m43 | -0.066 | Y_10 | -0.072 | Z_m18 | -0.088 |
| X_m62 | -0.074 | Y_m33 | -0.082 | Z_68 | -0.088 |
| X_m30 | -0.090 | Y_12 | -0.087 | Z_54 | -0.088 |
| X_42 | -0.097 | Y_45 | -0.090 | Z_58 | -0.091 |
| X_m14 | -0.111 | Y_m68 | -0.098 | Z_41 | -0.091 |
| X_m10 | -0.112 | Y_m38 | -0.101 | Z_63 | -0.110 |
| X_m31 | -0.119 | Y_20 | -0.123 | Z_33 | -0.111 |
| X_10 | -0.136 | Y_16 | -0.123 | Z_m10 | -0.113 |
| X_32 | -0.143 | Y_m26 | -0.126 | Z_m52 | -0.114 |
| X_m32 | -0.151 | Y_21 | -0.128 | Z_27 | -0.128 |
| X_m22 | -0.153 | Y_m36 | -0.145 | Z_18 | -0.131 |
| X_28 | -0.161 | Y_24 | -0.158 | Z_m15 | -0.142 |
| X_m13 | -0.179 | Y_57 | -0.180 | Z_m24 | -0.143 |
| X_m34 | -0.185 | Y_60 | -0.205 | Z_m46 | -0.180 |
| X_50 | -0.213 | Y_m3 | -0.210 | Z_m7 | -0.198 |
| X_m46 | -0.218 | Y_9 | -0.218 | Z_3 | -0.206 |
| X_0 | -0.269 | Y_m48 | -0.220 | Z_26 | -0.208 |
| X_26 | -0.275 | Y_m72 | -0.226 | Z_0 | -0.315 |
| **Component 2** | | | | | |
| X_41 | 0.250 | Y_m23 | 0.237 | Z_20 | 0.299 |
| X_17 | 0.244 | Y_m83 | 0.230 | Z_36 | 0.220 |
| X_14 | 0.243 | Y_m90 | 0.212 | Z_m34 | 0.212 |
| X_m20 | 0.230 | Y_44 | 0.206 | Z_25 | 0.197 |
| X_m16 | 0.218 | Y_m96 | 0.198 | Z_m16 | 0.193 |
| X_60 | 0.183 | Y_m103 | 0.180 | Z_0 | 0.179 |
| X_8 | 0.168 | Y_m66 | 0.153 | Z_56 | 0.176 |
| X_m64 | 0.166 | Y_26 | 0.150 | Z_75 | 0.176 |
| X_m57 | 0.159 | Y_m44 | 0.147 | Z_m4 | 0.151 |
| X_20 | 0.157 | Y_m52 | 0.147 | Z_m28 | 0.151 |
| X_56 | 0.148 | Y_50 | 0.141 | Z_61 | 0.139 |
| X_55 | 0.144 | Y_m54 | 0.140 | Z_39 | 0.135 |
| X_m11 | 0.126 | Y_m78 | 0.123 | Z_m9 | 0.134 |
| X_16 | 0.124 | Y_19 | 0.119 | Z_m44 | 0.124 |
| X_64 | 0.119 | Y_m105 | 0.115 | Z_34 | 0.119 |
| X_34 | 0.114 | Y_m17 | 0.114 | Z_m39 | 0.109 |
| X_19 | 0.112 | Y_m40 | 0.099 | Z_m31 | 0.108 |
| X_25 | 0.110 | Y_38 | 0.095 | Z_m29 | 0.101 |
| X_m41 | 0.099 | Y_m4 | 0.091 | Z_m38 | 0.099 |
| X_m60 | 0.088 | Y_54 | 0.081 | Z_3 | 0.097 |
| X_66 | 0.086 | Y_m12 | 0.078 | Z_35 | 0.095 |
| X_m69 | 0.082 | Y_17 | 0.073 | Z_40 | 0.091 |
| X_m18 | 0.076 | Y_53 | 0.034 | Z_m11 | 0.091 |
| X_m53 | 0.074 | Y_m84 | 0.029 | Z_m23 | 0.070 |
| X_m36 | 0.071 | Y_m20 | 0.029 | Z_m19 | 0.052 |
| X_54 | 0.070 | Y_m45 | 0.020 | Z_m22 | 0.047 |
| X_m52 | 0.068 | Y_m1 | 0.020 | Z_32 | 0.039 |
| X_m49 | 0.066 | Y_46 | 0.017 | Z_38 | 0.038 |
| X_m5 | 0.058 | Y_29 | 0.016 | Z_43 | 0.026 |
| X_m8 | 0.043 | Y_m7 | 0.015 | Z_8 | 0.012 |
| X_26 | 0.039 | Y_40 | 0.014 | Z_59 | 0.003 |
| X_48 | 0.025 | Y_14 | 0.008 | Z_m7 | -0.004 |
| X_49 | 0.023 | Y_m58 | 0.003 | Z_48 | -0.005 |
| X_4 | 0.015 | Y_m76 | 0.003 | Z_m1 | -0.011 |
| X_m58 | 0.009 | Y_m27 | -0.003 | Z_7 | -0.014 |
| X_22 | 0.008 | Y_45 | -0.009 | Z_4 | -0.021 |
| X_m42 | 0.008 | Y_m85 | -0.028 | Z_m33 | -0.031 |
| X_12 | 0.007 | Y_m39 | -0.031 | Z_41 | -0.032 |
| X_39 | -0.002 | Y_59 | -0.034 | Z_21 | -0.032 |
| X_m48 | -0.002 | Y_m59 | -0.037 | Z_44 | -0.038 |
| X_33 | -0.003 | Y_m2 | -0.044 | Z_m36 | -0.038 |
| X_45 | -0.004 | Y_57 | -0.047 | Z_m3 | -0.039 |
| X_38 | -0.015 | Y_m42 | -0.048 | Z_27 | -0.041 |
| X_40 | -0.022 | Y_35 | -0.066 | Z_74 | -0.044 |
| X_m62 | -0.024 | Y_58 | -0.066 | Z_m50 | -0.045 |
| X_m12 | -0.026 | Y_15 | -0.067 | Z_14 | -0.051 |
| X_m3 | -0.029 | Y_64 | -0.068 | Z_58 | -0.051 |
| X_m39 | -0.195 | Y_1 | -0.194 | Z_53 | -0.205 |
| X_9 | -0.255 | Y_m77 | -0.231 | Z_15 | -0.303 |

Notes: The term “m” represents “minus”. Each loading value is described in subjected to ascending orders.
